# Consistency and professionalism in balneology and medical rehabilitation

**Published:** 2018

**Authors:** Victor Lorin Purcarea

**Affiliations:** * “Carol Davila” University of Medicine and Pharmacy, Bucharest

Constant efforts of elite professionals in this important field are more and more, more known and, of course, more beneficial.

Thus, to highlight only two of the these remarkable actions, “*Integrative dimension of capitalizing the sanogenic resources from Techirghiol lake area*” National Conference with international participation, that took place at Techirghiol Balneary and Rehabilitation Sanatorium, at the beginning of this month, what should be mentioned is the marking of the 119 years of clinical research and maritime balneary management and especially the 45 years since the inauguration of Techirghiol Balneary and Rehabilitation Sanatorium.

The high scientific event, organized admirably and directed by the energetic and inspired “wand” of lawyer Elena Roxana Almăşan, PhD student and manager of the Sanatorium, was held in collaboration with the local and county decision makers and, among others, with the National Institute of Rehabilitation, Physical Medicine and Balneoclimatology, “Grigore Antipa” National Institute for Marine Research and Development, Romanian Society of Physical Medicine, Rehabilitation and Balneoclimatology, Romanian Society for NeuroRehabilitation, under the aegis of Romanian Medical Association and under the auspices of Medical Sciences Academy, “Carol Davila” University of Medicine and Pharmacy in Bucharest, and “Ovidius” University in Constanta.

**Fig. 1:**
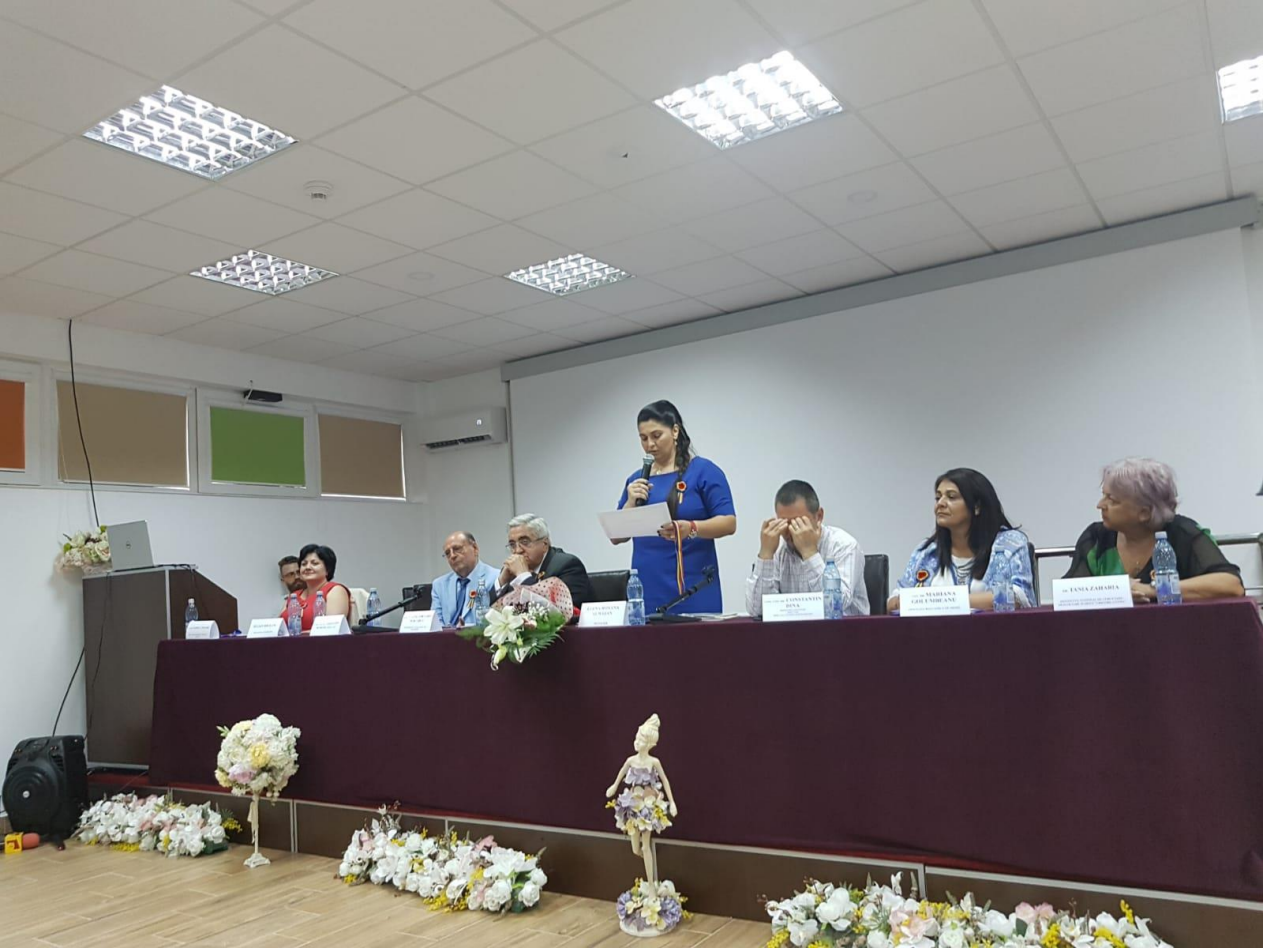
Presidium of “Integrative dimension of capitalizing the sanogenic resources from Techirghiol lake area” National Conference with international participation

The inaugural conference was held by the famous neurologist and psychiatrist, Prof. Dumitru Constantin Dulcan, MD, PhD, with the theme “*The rehabilitation function of the human brain*”.

A special moment of the Conference was the round table dedicated to the implementation of the main educational components belonging to the neurorehabilitation European curriculum in Romania, to which many important personalities in the field have participated: Prof. Dafin Mureşanu, MD, PhD, Prof. Ovidiu Băjenaru, MD, PhD, Prof. Bogdan Popescu, MD, PhD, Prof. Gelu Onose, MD, PhD, Prof. Cristina Daia, MD, PhD, Prof. Volker Hömberg, MD, PhD, Prof. Giorgio Sandrini, MD, PhD.

**Fig. 2:**
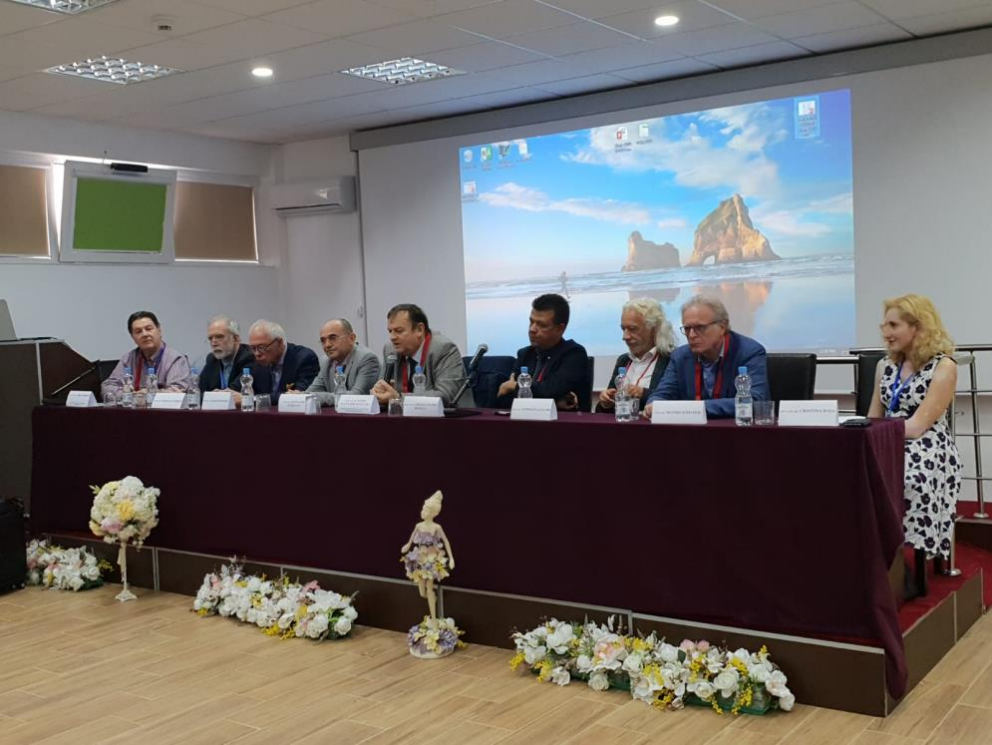
round table dedicated to the implementation of the main educational components belonging to the neurorehabilitation European curriculum in Romania

Another moment, with a powerful emotional impact, was the one of offering diplomas and medals to the employees, still working or retired, who were hired at the foundation of the Sanatorium, on August 1, 1973, and the evoking and homage paid to Cornelia Dărângă, MD, the first director of the institution (1973-1986).

**Fig. 3:**
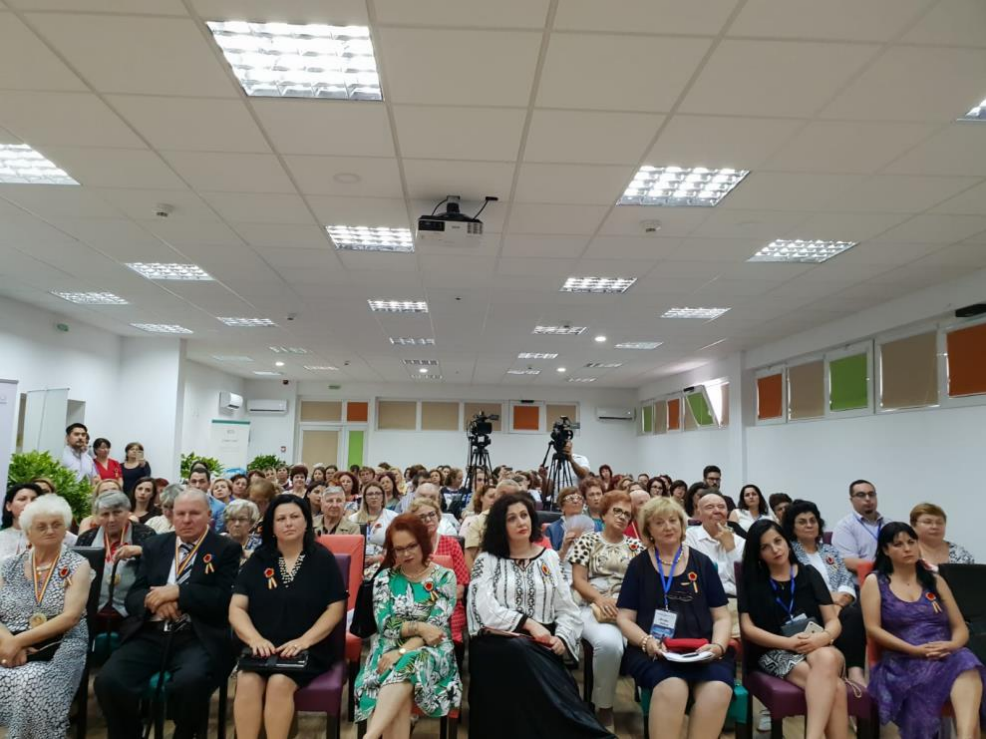
Employees of the Sanatorium, still working or retired, who were offered diplomas and medals

Among the materials offered to the participants, the following should be mentioned: the Abstracts published in a Special Issue of the valuable magazine “Journal of Medicine and Life”, *Techirghiol* magazine, *Arc peste timp* brochure and the anastatic edition of *Biodinamica lacului Techirghiol* by Ion Țuculescu.

Another remarkable event in this field took place this year in Aula Magna, Romanian Patriarchal Palace, under the patronage of the Academy of Medical Sciences and “Carol Davila” University of Medicine and Pharmacy in Bucharest, this being the 15^th^ National Conference of Balneology, and Medical Rehabilitation, organized by the National Institute of Rehabilitation, Physical Medicine and Balneoclimatology.

The dynamic and hearty manager of the Institute, Horia Lăzărescu, MD, PhD, managed to invite both the managers of the best spa resorts in Romania and the mayors of these resorts to this valuable scientific event.

**Fig. 4:**
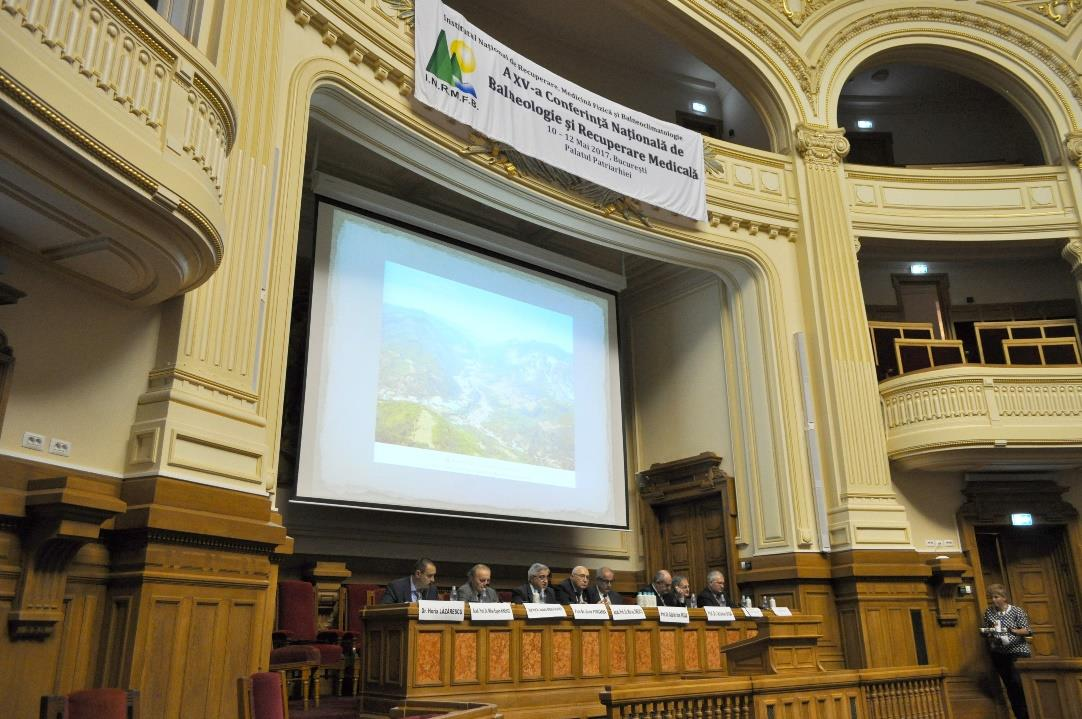
Presidium of the 15^th^ National Conference of Balneology, and Medical Rehabilitation, Aula Magna, Romanian Patriarchal Palace

The Conference had 12 scientific sessions and round tables, in which 87 scientific communications were presented and elaborated by 115 specialists on the following themes: “Novelties in balneology”, “Medical rehabilitation in neurological and cardiovascular diseases“, “Medical rehabilitation in rare diseases of children”, “Musculoskeletal ultrasound in monitoring the medical rehabilitation treatment”.

**Fig. 5:**
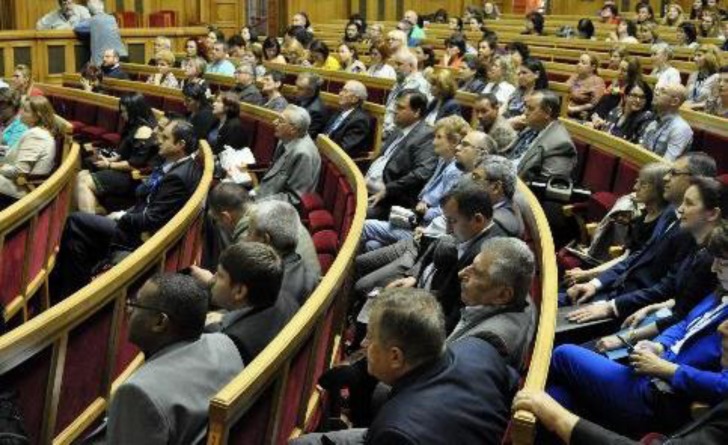
Participants at the 15^th^ National Conference of Balneology, and Medical Rehabilitation

On this occasion, the National Institute of Rehabilitation, Physical Medicine, and Balneoclimatology organized a special festivity, during which 15 spa resorts were awarded prizes for the quality of natural therapeutic factors, treatment centers, and the involvement of local public administration, as well as the therapeutical mineral waters. The excellence diplomas and the medals of honor were offered to the mayors of the spa resorts and the managers of the sanatoriums or treatment centers.

“Carol Davila” University Press of “Carol Davila” University of Medicine and Pharmacy in Bucharest was very much appreciated at this remarkable event, being present with specialty bookstands, among which the Romanian version of “*Recuperarea Medicală de fază acută*”, edited by Prof. Adriana Sarah Nica, MD, PhD.

**Fig. 6:**
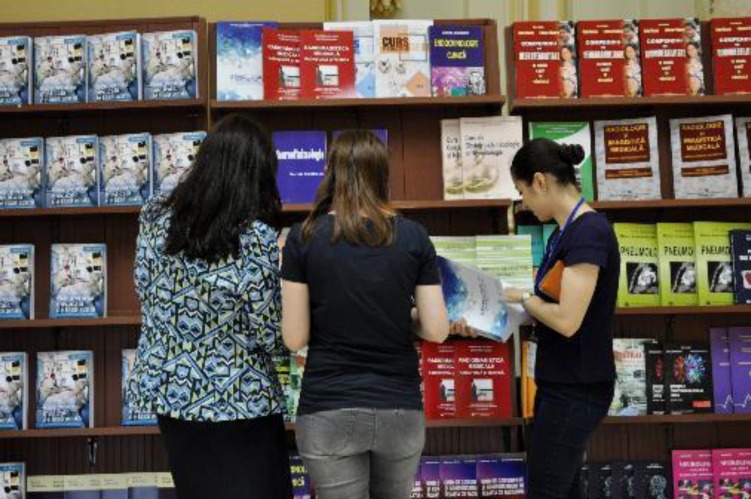
Bookstands of “Carol Davila” University Press, Bucharest

**Fig. 7:**
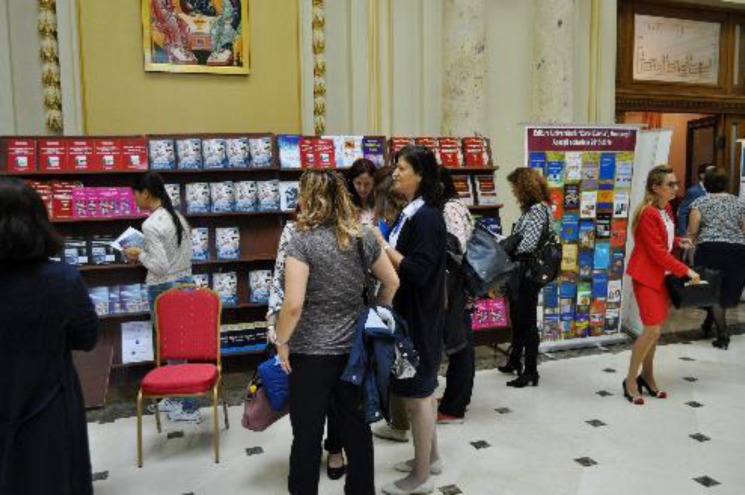
Participants interested in the books presented by “Carol Davila” University Press, Bucharest

**Executive Editor Professor Eng. Victor Lorin Purcarea, PhD**

